# Current and Emerging Techniques for High-Pressure Membrane Integrity Testing

**DOI:** 10.3390/membranes8030060

**Published:** 2018-08-09

**Authors:** Eddy R. Ostarcevic, Joseph Jacangelo, Stephen R. Gray, Marlene J. Cran

**Affiliations:** 1 Institute for Sustainable Industries and Liveable Cities, Victoria University, Werribee 3030, Australia; eddy.ostarcevic@vu.edu.au (E.R.O.); stephen.gray@vu.edu.au (S.R.G.); 2 Stantec, Washington, DC 20005-3957, USA; joseph.jacangelo@stantec.com

**Keywords:** reverse osmosis, nanofiltration, membrane integrity

## Abstract

Ideally, pressure driven membrane processes used in wastewater treatment such as reverse osmosis and nanofiltration should provide a complete physical barrier to the passage of pathogens such as enteric viruses. In reality, manufacturing imperfections combined with membrane ageing and damage can result in breaches as small as 20 to 30 nm in diameter, sufficient to allow enteric viruses to contaminate the treated water and compromise public health. In addition to continuous monitoring, frequent demonstration of the integrity of membranes is required to provide assurance that the barrier to the passage of such contaminants is intact. Existing membrane integrity monitoring systems, however, are limited and health regulators typically credit high-pressure membrane systems with only 2 log_10_ virus rejection, well below their capability. A reliable real-time method that can recognize the true rejection potential of membrane systems greater than 4 log_10_ has not yet been established. This review provides a critical evaluation of the current methods of integrity monitoring and identifies novel approaches that have the potential to provide accurate, representative virus removal efficiency estimates.

## 1. Introduction

**Figure 1:**
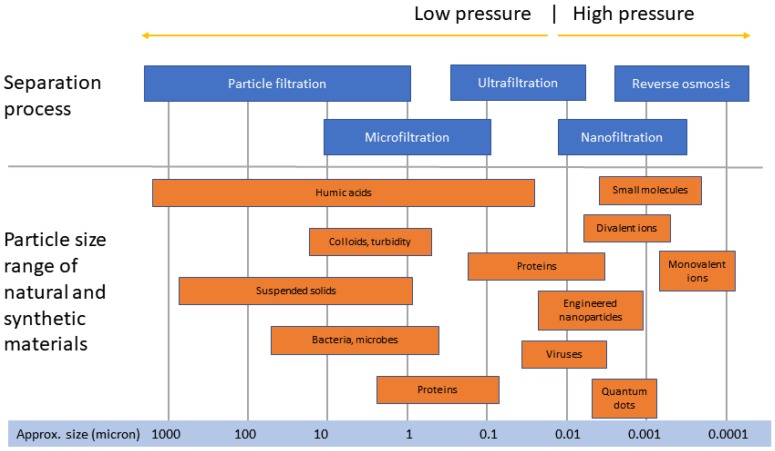
Schematic representation of membrane barrier performance.

High-pressure nanofiltration (NF) and reverse osmosis (RO) membranes are widely used for water reclamation and desalination [1,2,3]. The mechanism of water transport in these high-pressure membrane systems is based on solute transport by diffusion through the nonporous active membrane layer [4,5]. These membranes are, therefore, capable of rejecting particles, bacteria and pathogens, and dissolved ions including salts as illustrated in Figure 1. In reality, however, NF and RO membranes are not complete barriers to these contaminants [6] and over time the integrity of membrane systems may be compromised due to a range of breaches that can develop in the system. Membrane breaches can occur as a result of: damage to the membrane layer as a result of chemical or biological degradation and particulate abrasion; delamination of the skin layer from the supporting layer; telescoping; and failures of O-rings, gaskets, connectors and other fittings [7,8,9,10,11,12,13]. In addition to these ‘in service’ failure modes, manufacturing defects such as incomplete glue-lines can affect the integrity of membrane elements and depending on the quality control processes employed, these are usually detected before membranes are shipped to customers.

In order to ensure the quality and safety of water treated by NF or RO membranes, the integrity of the entire system must remain intact over the service life of the elements. Moreover, system operators must be able to provide evidence of the integrity to satisfy regulatory requirements on a regular basis [14]. Several integrity monitoring techniques have been developed for these systems and they are broadly classified into direct and indirect techniques [6,15]. In general, direct methods assess the integrity of membrane elements by the application of a pressure-based test whereas indirect methods use a surrogate parameter measured in the permeate for assessing the integrity of the whole membrane system [6,16,17]. Direct tests are usually performed offline on individual elements but indirect tests can be performed on membranes during service and have a wider scope for online continuous monitoring. Challenge tests are a type of indirect technique where a surrogate species is introduced into the feed water and the removal efficiency is determined by the measurements of the surrogate species in the permeate [16,17].

Several reviews of high-pressure membrane integrity monitoring techniques have been published with a focus on existing, widely accepted methods [15,18,19,20]. These methods are primarily focused on common spiral-wound NF or RO membranes, although there are examples of integrity monitoring of hollow-fiber NF membranes [21]. In order to fully realize the rejection capabilities of high-pressure systems, new, robust techniques that can be implemented in real-time and online are required. This paper presents a review of existing integrity tests and focuses on emerging techniques that have the potential to demonstrate the rejection capabilities of spiral-wound high-pressure membrane systems online. This is followed by a discussion of current methods and potential methods that show promise for future development.

### 1.1. Log Reduction Value

For membrane processes, the rejection or removal efficiency of a given parameter or surrogate is reported as a log reduction value (LRV) that is calculated according to the following formula:

LRV = log_10_ (*C*_f_/*C*_p_) (1)

where *C*_f_ is the feed concentration of the chemical or surrogate species and *C*_p_ is the concentration detected in the permeate [22].

Although LRVs are widely reported, their determination is limited by the feed concentration and the detection limits of the chemical or surrogate in the permeate. Nevertheless, the aim of a challenge test is to report a removal efficiency based on the feed concentration of the surrogate, so it is also important to be consistent with dosing of surrogates for a given system to enable direct comparisons of LRVs over time. The principle of LRV applies to any integrity monitoring technique that has the capacity to enumerate the feed and permeate concentrations of the surrogate and is adopted as the industry standard for the verification of membrane integrity.

### 1.2. The Target—Poliovirus

Conventional treatment incorporating membrane filtration plus disinfection practices have been successful in preventing the spread of waterborne pathogens to the public. However, monitoring for all bacterial, viral and protozoan species would be extremely expensive and a drain on operational resources. For this reason, a surrogate species in the RO feedwater should be identified and monitored [23]. Larger bacterial and protozoan pathogens are typically eliminated by unit operations employed upstream of high-pressure membranes and via size exclusion but not necessarily for viruses that are considerably smaller. The performance of advanced water treatment operations that incorporate high-pressure membranes is, therefore, critical to effective virus control.

Monitoring for viruses is challenging and requires specialist skills and apparatus that must be undertaken offline in a laboratory with results available sometime after the samples were taken, usually several days. This does not equate to timely monitoring of membrane integrity, particularly in the event that a membrane or system fails the integrity test. Consequently, the challenge is to select a non-microbial surrogate can mimic the target virus in terms of size and shape. Viruses are classified into families and then genera, based on many criteria, including physical and biological properties. *Picornaviridae* are the smallest family of RNA viruses [24], with picornaviruses divided into four groups [25]: poliovirus (3 types), coxsackievirus (30 types), echovirus (34 types) and enteroviruses (68 to 71 types).

An important characteristic of enteroviruses is their resistance to high and low pH and relative stability over a pH range of 3–5 for up to 3 h [25]. Alcohol at 70 vol %, 1% quaternary ammonium compounds and other common laboratory disinfectants are not effective against enteroviruses which are reported to be insensitive to detergents that otherwise destroy lipid containing viruses [25]. In theory, high-pressure RO and some NF membranes should exclude virus size particles providing they are intact. The poliovirus is one of the smallest of the RNA viruses with a shell (capsid) between 25 and 30 nm in diameter [24,25] and is therefore difficult to remove by size exclusion membrane processes. The ability to demonstrate that high-pressure membrane systems can reject such virus size particles is therefore crucial and challenge testing with similarly sized non-microbial surrogates is one possibility providing that the surrogate can be detected at very low concentrations to meet the target of at least 99.99% (4 log_10_) removal in accordance with the US EPA Long Term 2 Enhanced Surface Water Treatment Rule [26].

### 1.3. The “Ideal” Integrity Test

In order to evaluate individual integrity monitoring systems, it is important to define the characteristics of an “ideal” system. In general, an ideal system is one that can accurately measure the ability of a system to reject pathogens in a reasonable time so that the risk of these pathogens entering the product water is minimized or eliminated. The cost of performing the integrity test should also be considered as these costs may be passed on to consumers. Table 1 presents a summary of some suggested key criteria that any potential monitoring system should be measured against.

## 2. Direct Integrity Monitoring

Direct monitoring techniques are used to measure the integrity of single membrane elements or very small RO systems. These tests are primarily pressure-based techniques that are used to detect defects such as pinholes in membrane sheets and glue-line failures. Vacuum decay and pressure decay tests are the most common direct monitoring techniques applied to NF and RO membranes [7,26,27,28] and the capital costs for instrumentation to measure these decay tests is relatively low. However, these techniques are not able to detect holes of the size that can permit the passage of virus sized particles except at very high-pressures that could potentially result in damage to membrane elements. Moreover, these tests must be performed when the membrane filtration system is offline.

### 2.1. Vacuum Decay Testing

**Figure 2:**
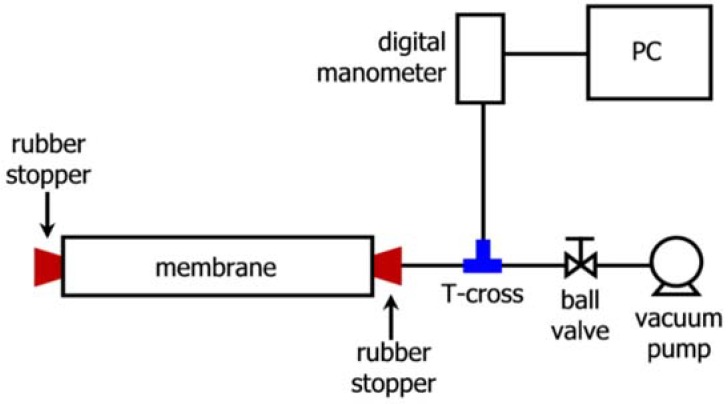
Schematic illustration of vacuum decay test (VDT) apparatus.

In a vacuum decay test (VDT), the permeate tube of a membrane element is sealed and a vacuum is applied to the tube. A schematic representation of VDT equipment is shown in Figure 2. The basic procedure in accordance with ASTM D3923 [27] and ASTM D6908 [28] involves soaking the membrane element for a period of time in RO permeate water (usually overnight), draining the membrane module for a defined period of time (usually an hour) and sealing one end of the permeate tube using a leak tight cap. As shown in the schematic, Figure 2, the system can be integrated with a digital manometer and computer to record the VDT data and generate the decay curve.

**Figure 3:**
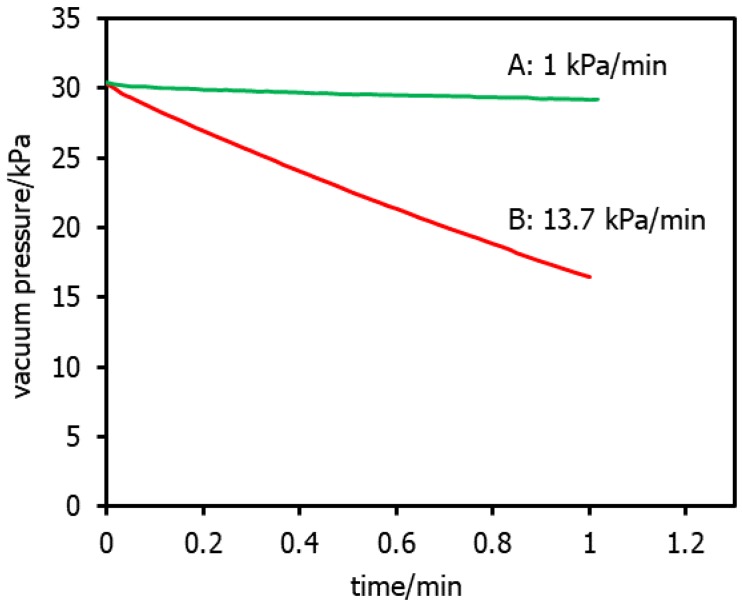
Examples of VDT results from intact (A) and compromised (B) RO membrane elements.

The rate of decay of the applied vacuum pressure is monitored over a set period of time. For RO elements, a decay rate of >10 kPa min^−1^ represents a significant membrane defect and this test is typically performed on elements by the manufacturer prior to sale [6,27,29]. This method can be applied to a single element or a complete pressure vessel containing several elements for small scale RO systems [30]. Figure 3 shows typical VDT results from two different commercially available RO membranes with one intact and one compromised. The vacuum pressure of the intact membrane shows only a slight decline whereas the compromised element shows a rapid decline indicative of a gross defect.

The VDT method is generally only applicable for the detection of mechanically damaged membrane elements or damaged O-rings and will not detect chemical impairments that create much smaller inconsistencies in the membrane. Therefore, this method is only useful as a screening procedure to detect significant defects in RO systems. However, some reports suggest that VDT results correlate well with virus and total organic carbon (TOC) rejection by RO membranes and it is therefore a potentially useful integrity screening procedure for RO membrane elements [6]. Since the test is applied directly to membrane elements, the VDT cannot be performed when filtration is taking place [16] and thus does not enable real-time membrane integrity monitoring in the strictest sense but could allow frequent monitoring to identify slow degradation of system integrity.

### 2.2. Pressure Decay Testing

In a pressure decay test (PDT), the feed and concentrate ends of a membrane element are sealed and compressed air is introduced. In this case, the permeate side remains open and the pressure decay is measured over time. This test is particularly useful for measuring the integrity of low pressure hollow fiber MF and UF membranes [26,28,31] and although the PDT is not common for NF and RO membranes, there are examples of patented methods for PDT of spiral wound modules on wet or dry elements [32]. In an example of a PDT trial on an entire stage of RO vessels for a small scale RO application [33], the membranes were drained, the permeate side was pressurized to 600 kPa (87 psi) and the pressure decay was monitored over a 10-min period. The study concluded that this method was sensitive in detecting breaches but was not practical for full-scale systems because the entire element array must be drained prior to the test. Other examples have demonstrated the use of automated PDT on RO systems for direct potable reuse applications [13,34]. In practice, the main aim of the PDT for RO in most cases is to determine an LRV for relatively large protozoa and this only requires low pressures. Moreover, this technique, that has similarities to the VDT, has the potential to induce mechanical failures such as telescoping in spiral wound elements. Telescoping can occur during a PDT as a result of the charging and discharging of compressed air from an RO element [13] although this can be prevented by the use of anti-telescoping devices [35].

## 3. Indirect Integrity Monitoring

Indirect monitoring techniques measure the rejection of chemical surrogate species that are naturally present in the feed water in order to report the permeate quality. Some indirect monitoring techniques can be performed online but often the species being measured are smaller than virus-sized particles such as salt and organic carbon. In these cases, the compounds may not be as effectively rejected by RO membranes and measurement systems are therefore only capable of validating to low LRVs. Other indirect monitoring techniques include challenge tests where a surrogate species (chemical, nanoparticle, bacteriophage, etc.) is introduced into the feed water at a known concentration in order to evaluate the amount of the surrogate that can pass into the permeate. In any case, indirect monitoring based on the rejection of a chemical species results in a more conservative measurement of the system LRV and is based on the ability of the membrane to exclude the chemical rather than virus-sized particles.

### 3.1. Naturally Occurring Substances

#### 3.1.1. Particle and Turbidity Monitoring

A typical particle monitoring device measures changes in light transmittance through a fixed volume of permeate as a result of particles blocking the light source. The minimum particle size these monitors can detect is ca. 500 nm, which is considerably larger than any particle that could breach an NF or RO system. As such, particle monitors are generally used in MF and UF systems rather than NF and RO systems. In addition, air bubbles can interfere with the signal output causing erroneous results.

Online particle monitoring for RO is generally not recommended due to the high removal of particles by MF/UF prior to RO treatment [6]. However, not all RO treatment processes utilize MF/UF pre-treatments so in general, the resolution for these particle monitoring systems is dependent on the type of pretreatment. In addition, particles large enough to be detected in particle counters may be present in RO permeates in events such as O-ring or permeate interconnector damage that are considered as gross integrity failures.

The principle of turbidity monitoring is similar to that of particle monitoring whereby the passage of light through a sample is measured and related to the concentration of suspended particles. Typical nephelometric sensors also suffer from similar disadvantages including low sensitivity and interference. Laser turbidimetry, however, can reportedly measure much lower turbidity values [36,37] but this is limited to particles larger than ca. 1000 nm in diameter that is on the order of thirty-fold larger than the smallest pathogens of concern at between 25 and 30 nm.

#### 3.1.2. Total Organic Carbon

Total organic carbon monitoring involves the oxidation of carbon and the detection of carbon dioxide generated during the process. Pre-treatment for NF/RO membranes should exclude all organic particulates and therefore the TOC is equivalent to the dissolved organic carbon content. The measurement of TOC is reported to be more sensitive than conductivity testing and particle counting [6] and although the measurement of TOC is perhaps the closest to instantaneous integrity monitoring, typical TOC measuring devices are prone to drift. Moreover, TOC is not representative of virus-sized particles as it is a measure of water soluble compounds that are generally much smaller. Additionally, their chemical structure can be more flexible than viruses and this combined with their smaller size, may allow passage through membranes. As a consequence, TOC monitoring will generally provide a more conservative LRV because the soluble compounds can be transported across the membrane into the permeate where virus-sized particles would otherwise be rejected. A major limitation of this test and most other indirect techniques is related to the sensitivity of the instrumentation. Given that the sensitivity of TOC is limited due to the relatively low RO/NF feed concentrations (around 10–20 mg/L) and the lower resolution of the instruments (around 0.15 mg/L), TOC monitoring can only provide a rejection of around 99.75% (2.6 log_10_). A high sensitivity instrument is ideal to measure the low TOC found in the permeate, however, it is unlikely that the same instrument could reliably measure the higher levels of TOC in the feed, thus necessitating the need for two complex and expensive instruments. This would limit the use of this technique to large treatment plants producing large volumes of treated water for direct or indirect potable use applications. Additionally, changes in organic and inorganic carbon dioxide compositions within the feed, as well as changes in temperature and fouling conditions may result in higher or lower than anticipated organic carbon rejections resulting in inaccurate integrity results. Nevertheless, full-scale online TOC monitoring has been implemented at the NEWater plant in Singapore to monitor the integrity of an RO system installed for indirect potable reuse applications with a Sievers 820 online TOC instrument with a detection limit of 0.15 mg/L installed at this facility [38].

#### 3.1.3. Sulphate Monitoring

The monitoring of naturally occurring sulphate levels can potentially be implemented online and can reportedly provide high resolution, provided the feed concentration is consistent and sufficiently high [39]. Measurement is typically performed by ion chromatography and a resolution of 3 log_10_ removal has been reported with a feed sulphate concentration of 140 mg/L and a detected permeate sulphate concentration of 0.1 mg/L [29]. The measurement of sulphate can be achieved ex-situ and reported quickly but this test does not provide real-time information on the integrity of the membrane system and can become difficult where multiple trains operate in parallel.

As a soluble chemical species, sulphate is much smaller than the smallest pathogens such as viruses, so this technique offers only a conservative evaluation of membrane integrity. Moreover, the mechanism of transport of sulphate through a membrane would be expected to differ significantly than that of a solid virus particle. In addition, biological species entrained in biofoulants on the membrane surface can utilize the oxygen under anoxic conditions so the measured sulphate concentration in the feed can be reduced thereby altering the LRV. Scale forming compounds such as BaSO_4_ and CaSO_4_ can also alter the measured permeate sulphate concentration as well as adversely affect membrane performance and prematurely trigger the need for a clean-in-place procedure to restore performance. These issues represent a significant limitation to the efficacy of this technique.

#### 3.1.4. Electrical Conductivity

Electrical conductivity (EC) is generally used as an indicator of membrane integrity, however, the sensitivity of such measurements can be grossly distorted through an array. Temperature, feed concentration, pressure, position along an array and fouling all have an effect on permeate EC and slight fluctuations in permeate EC can mask compromised membranes. Conductivity testing can be implemented online or via intermittent probing with the latter providing higher sensitivity than the online technique [6]. This is useful when the feed water contains a relatively high concentration of salt that is efficiently rejected by RO [18]. Typically, damage to components of RO systems such as an O-ring can cause a spike in permeate conductivity that can indicate a leak.

Due to its ease of implementation, conductivity monitoring is a common method for detection of integrity breach at full-scale for NF and RO systems. However, the resolution of this method is low and will provide only up to 2 log_10_ removal when feed EC is around 20,000 µS/cm and permeate EC is 200 µS/cm. Electrical conductivity readings are subject to fluctuations as a result of temperature, ionic species in the feed as well as membrane fouling. Ionic speciation can have a significant impact on electrical conductivity with predominantly monovalent species having a higher TDS to EC ratio whereas a greater proportion of divalent ions in the feed will reduce the TDS to EC ratio. This can be significant in industrial applications that have a high degree of variation in discharged wastewater characteristics and have the potential to inadvertently introduce bias in the LRV result in specific circumstances.

#### 3.1.5. Periodic Testing

Periodic monitoring methods include conductivity profiling, conductivity probing and UV-254 probing. Conductivity profiling involves sampling permeate from each pressure vessel and analyzing the conductivity, allowing this method to detect a breach in a particular pressure vessel. Conductivity probing is widely used to determine the location of a leak within a pressure vessel and is usually combined with conductivity profiling [40,41]. Probing for UV absorbance at the 254 nm wavelength has also been reported [33] but this method is limited because it can only achieve low resolution which depends on the concentration of UV active species present in the RO feed water. Similar to EC monitoring, a spike in the measurement of the monitored species will clearly indicate a breach in membrane integrity [2].

### 3.2. Challenge Testing

#### 3.2.1. Dyes and Tracer Chemicals

Chemical tracers have been used widely to study flow patterns in a range of water and soil systems [42,43]. These include the use of fluorescent dyes such as fluorescein [17,43,44,45] and rhodamine WT (RWT) [42,43,45,46] and synthetic food dyes such as Brilliant Blue FCF [47,48]. The fluorescent dyes are particularly useful as water tracers, due to their high sensitivity of detection and selectivity when contained in systems with other fluorescent compounds. Detection limits for these compounds are in the region of 100 ng/L levels so only small amounts are needed in the feed [45]. Rhodamine WT is a non-toxic fluorescent chemical that was specifically developed as a water tracing dye [49,50]. As such, it is reported to be stable under a range of saline conditions [46,51] and has shown potential for use in RO integrity monitoring [8] with a minimum detection limit of approximately 10 ng/L [44]. Instrumentation for measuring fluorescent tracers, including online commercial probes for measuring RWT, are readily available [52,53].

Integrity monitoring using RWT has been widely reported for RO systems in order to assess virus removal or to determine LRVs [8,9,14,18,26,28,54,55,56,57,58]. In addition to its high sensitivity, RWT was also reported to be stable under a range of environmental conditions and not to be readily adsorbed onto membrane surfaces. However, the fluorescence can be affected by the presence of chlorinated oxidizing agents, UV light and temperature. Under full-scale and pilot testing using RWT, LRVs of >4 log_10_ have been achieved for several RO membranes [54,57,58]. As a soluble chemical, however, the correlation between RWT and solid particles such as viruses and other pathogens is limited, with RWT providing a conservative estimate of pathogen LRV. Table 2 presents a summary of several RWT field or laboratory tests and LRVs obtained. In most cases, high LRVs can be achieved with low concentrations of RWT in the feed.

#### 3.2.2. Spiked Integrity Monitoring

A relatively new technique, the spiked integrity monitoring (SIM) test can be performed intermittently and online by dosing or injecting a challenge species into the feed [29,59]. In these examples, powdered activated carbon (PAC) was used as the challenge species and was detected using particle counters with relatively high LRVs achieved depending on the dose of the PAC added to the feed. Although this technique is applicable to MF/UF membranes, it could be applied to NF and RO systems providing the PAC is sufficiently small and monitoring systems are available to detect small particles. However, the potential for sub-micron PAC particles to agglomerate with biofoulants can result in artificially high LRVs, premature fouling, and increased biological activity on the surface of the PAC particles.

#### 3.2.3. Pulse Integrity Testing

The pulse integrity test (PIT) is similar in principal to the SIM and involves the addition of a short pulse of a highly rejected chemical species such as sulphate salts (i.e., MgSO_4_ or Na_2_SO_4_) [60,61]. The sulphate is monitored in the permeate over time then compared to known profiles representing intact and compromised membranes. Defects such as holes and glue-line faults present distinct sulphate profiles which can also be attributed to different locations in a membrane module. Although this protocol appears promising, it does not provide continuous security against membrane breaches, however, it does present a potentially useful tool to characterize the failure mode and location of a breach in a membrane array.

This technique also has the potential to be used online and in real-time providing the test is calibrated for each system although this would be complicated, time consuming and expensive for large installations. There is also a potential to develop this technique further using other tracer species such as RWT or even particulate surrogates that could increase the detection limits and also enable a concurrent LRV determination. However, as previously discussed, the limitations of using sulphate as a surrogate include contributing to biological fouling and chemical scaling. Both of these conditions would reduce the sulphate available to migrate across a membrane and would artificially improve the apparent LRV. Unlike a challenge test, the membrane is only subjected to the pulse for a very short period minimizing the contact and potential absorption on the membrane surface.

#### 3.2.4. Microbial Surrogates

Microbial surrogates include the MS2 bacteriophage that is widely adopted as a substitute for pathogenic viruses [14] and is the recommended microbial surrogate for enteric viruses [26]. It is often used in membrane integrity testing due to its similar size (22–29 nm [62]), shape and composition to some common enteric viruses [63,64,65,66]. Standard methods or protocols for performing challenge tests using MS2 seeding have been developed and the results of pilot and full-scale tests are widely reported [9,14,18,26,63,67,68,69,70,71,72]. In one example, an LRV > 5 was reported for the removal of MS2 by intact RO membranes [63]. Although widely implemented, this technique has some limitations for application in full-scale plants including high costs, sample preparation and preservation and the potential for false positive results [17]. The use of this testing protocol to determine the integrity of an operational high-pressure membrane system is limited by this risk and implementing this testing procedure online is not feasible. In addition, enumerating the MS2 bacteriophage must be performed by a specialist laboratory and this time-consuming assessment does not provide for a real-time integrity evaluation of the operating membrane plant.

#### 3.2.5. Non-Microbial Nanoparticle Surrogates

The use of non-microbial surrogates removes some of the risk involved in performing challenge tests with high concentrations of live bacteriophage. Other than fluorescent dyes, non-microbial surrogates include fluorescent latex micro- and nano-spheres [8,54,58,63,70,73,74], gold nanoparticles [74] and magnetic nanoparticles [75,76] and other readily detectable particles [77]. These particles offer the potential for real-time and online monitoring which is one of the key criteria for the ideal NF/RO integrity test.

Most nanoparticle challenge tests do not consider the effects of solubility, surface chemistry, agglomeration and other factors that may influence rejection. For example, some nanoparticles have been reported to aggregate and foul the surface of membranes [78] and this can improve the rejection of similar sized particles resulting in higher LRVs than clean membranes, thereby providing an overestimation of system integrity. Other disadvantages include the costs associated with the production of fluorescent nanoparticles on a large scale. Although fluorescence detection instruments are widely available and relatively inexpensive, fluorescent nanoparticles can be very expensive to synthesize and large quantities would be required for full-scale operations. Fluorescence can also be quenched in the presence of salt, organic species, oxidants and several other chemicals and under certain environmental conditions. In addition, depending on how the fluorescent markers are attached to the latex particles, they can leach from the surface into the surrounding carrier solution. This can lead to detection of the dye marker in the permeate which would otherwise reject the intact particle, thus biasing the LRV determination. The fate and toxicity of synthetic or engineered nanomaterials is relatively unknown with the exception of common inorganic nanoparticles such as TiO_2_ [79].

Magnetic nanoparticles are an example of a particle that can potentially be recovered and reused in the case of UF integrity testing using 35 nm Fe_3_O_4_ particles [76]. Although this report suggested the technique was suitable for large-scale plants, the instrumentation to detect these particles would add considerable cost to existing and new plants. Magnetic resonance relaxometers used to detect magnetic nanoparticles are commercially available as bench-top instruments, however, the relaxometer and magnetic resonance scanner are impractical due to their high cost that results principally from the large magnets employed and the lack of miniaturized electronic components [80]. Other metal-based surrogates include silver nanoparticles which have shown some promise for integrity monitoring with particles of 62 ± 10 nm used to challenge used RO membrane elements [81]. In these tests, the feed concentration was > 8 mg/L and the maximum LRV obtained was 2.57 log_10_ although higher LRVs for UF and MF membranes have been reported for similar particles [19]. In addition, the detection technique used was inductively coupled plasma-optical emission spectrometry which restricts this method to offline analysis in a laboratory environment.

## 4. Integrated and Multi-Parameter Monitoring Systems

A range of other techniques for monitoring the condition of high-pressure membrane systems have been reported. Some of these techniques offer the potential for real-time monitoring while others are more qualitative and require integration with other instruments. Online hybrid systems can offer multiple detectors in a single unit that can increase the sensitivity of these units and reduce the need for separate instrumentation.

### 4.1. TRASAR^®^ Testing

The TRASAR^®^ system is based on the use of a fluorescent chemical with antiscalant compounds in order to monitor and control antiscalant dosing in RO systems and to prevent overdosing [82]. Monitoring the fluorescent marker that is part of the antiscalant chemical can be used as a potential integrity monitoring system. Fluorescence is measured in the permeate with LRVs up to 6 log_10_ when the chemical is added in a spiked dose and 2 log_10_ when added with antiscalant [18,72]. In principal, the system is similar to that of the dye test except that in the case of the TRASAR^®^ system, the fluorescent compound is a larger molecule than typical fluorescent dyes with a molecular weight ca. 610 Daltons. It would be expected that the sensitivity of TRASAR^®^ antiscalant would be lower than a dye such as RWT, so much higher concentrations would be needed to achieve an LRV of 6 log_10_. Similar to other fluorescent dyes, the use of the TRASAR^®^ fluorescent chemical compound could potentially detect integrity breaches from chemicals of concern such as endocrine disruptors that are smaller than virus particles, thereby extending the capabilities of the integrity test beyond microbial pathogens to chemical surrogates.

### 4.2. Small Sensor Cell Membrane Testing

In a small sensor cell membrane test, a micro-sieve sensor membrane is placed in the permeate side of the crossflow stream of a high-pressure system. A change in the TMP of the sensor membrane is detected as an integrity breach but it can take more than one hour to detect even a very small breach. In addition, the system is sensitive to the permeate flux, so to overcome this a second sensor membrane can be added [83]. This technique has shown promise at both the bench- and pilot-scale but a limitation of using this technique is the long delay before an integrity breach is detected.

### 4.3. Binary Gas Integrity Testing

The binary gas integrity test (BGIT) measures the diffusion of a select pair of gases injected into the feed of a low-pressure membrane system. For a membrane with an integrity breach, the low permeating gas is detected in the permeate using mass flowmeters and the composition determined using infrared spectroscopy [84]. This technique is reported to be highly sensitive and has been well correlated to virus removal [85]. However, the sensitivity can be limited by the minimum detectable excess flow and a range of intrinsic membrane factors can influence the measured gas diffusion rate. In this case, normalization to a baseline measurement would be necessary to compare membrane performance over time. In addition, the need for multiple gases and detectors limits the applicability and practicality of this technique. There may be some potential to apply this technique to high-pressure membranes at the small scale but the complexity associated with larger systems would generally preclude its adoption.

### 4.4. ZAPS LiquID Station

The zero angle photo-spectrometry (ZAPS) LiquID Station offers real-time water quality monitoring based on an entirely optical system utilizing three simultaneous techniques to measure a wide range of water quality parameters [86,87]. It uses absorption, fluorescence and reflectance measurement techniques with a single sensor, which eliminates the differential drift of multi-sensor devices. In addition, it uses no reagents and is self-cleaning and self-calibrating. It is typically used to monitor source water, drinking water treatment and distribution systems, wastewater treatment and re-use and industrial water systems [88,89,90]. The parameters it measures include TOC, UV254 transmission, specific UV absorbance, nitrate + nitrite, turbidity, biochemical oxygen demand (BOD) and chemical oxygen demand (COD). The detection ranges of the TOC and BOD alone can enable LRVs of ca. 5.7 and 6.5 log_10_ respectively.

In addition to the monitoring of standard indicator compounds present in the water sample, the system is capable of indirect detection of *E. coli*. Since enteric pathogens cannot occur without the presence of enteric bacteria, testing for these bacteria can be used to monitor pathogen risk. The ZAPS system detects tryptophan that is a highly fluorescent amino acid that bacteria and other living microorganisms utilize to manufacture proteins. The *E. coli* bacterium maintain high concentrations of tryptophan as part of their cellular structure so this chemical can, therefore, be used as a biomarker to detect their presence. However, tryptophan is a common amino acid in many proteins and is not unique to *E. coli* so this may limit its sensitivity for detecting this species.

## 5. Emerging Techniques

### 5.1. Pathogen Detection Systems

The ability to detect and quantify potentially hazardous pathogens in food and water is increasingly important to prevent and minimize the proliferation of infectious diseases [91]. The development of biosensors has grown rapidly over the past few decades with a range of devices available commercially and as prototypes [92,93,94]. Some of these show promise for the future development of membrane integrity monitoring techniques where the removal of viruses and other pathogens is critical.

#### 5.1.1. BioSentry Device

The BioSentry device is a commercially available instrument that can detect chemical and biological contamination in water and report detected problems continuously in real-time with online capabilities. The device measures unique bio-optical signals of particles and compares them to those in its library to identify microbial contaminants such as *E. coli*, *Giardia* and *Cryptosporidium* [95]. In addition to microbial species, the device is reported to detect chemical contamination in drinking water so it has the potential to be adapted to monitor membrane integrity. The system uses multiple sensors to measure a range of quality indicators and also uses a multi-angle light scattering detector at 660 nm to determine the size, shape and internal structure of detected particles. However, the current configurations of the system limit size analysis to particles greater than 400 nm [96] and that is an order of magnitude larger than the required resolution needed to detect viruses with an average size between 25 and 30 nm.

#### 5.1.2. Real-Time Polymerase Chain Reaction Monitoring

The real-time polymerase chain reaction (RT-PCR) technique is based on the detection of part of the viral genome [62]. Although the technique is relatively fast, it requires specialized sampling, testing and analysis and is therefore an expensive test that at present must be performed offline in a laboratory environment. In addition, the technique cannot differentiate between infectious and non-infectious viruses and this can complicate the results [97].

#### 5.1.3. Evanescent Wave Fiber Optic Sensors

Evanescent wave fiber optic sensors (EWFOS) use a laser spectrofluorometer to detect a laser derived evanescent wave that is excited over a sample [98,99,100,101]. These sensors can be highly customized to detect a range of pathogens and can be integrated into existing systems enabling the potential for online testing using flow-through systems. Although these EWFOS systems are currently susceptible to interference due to complex background matrices, it is expected that future EWFOS could be increasingly sensitive measuring concentrations as low as 1 CFU/mL [101]. However, this may not be sensitive enough to measure low permeate concentrations to provide a high LRV.

#### 5.1.4. Surface Generated Acoustic Wave Biosensors

In a surface generated acoustic wave (SGAW) biosensor, metal electrodes mechanically generate acoustic waves through a substrate such as a liquid medium [102]. Biochemical interactions with target pathogens result in changes in the acoustic wave that are detected and then analyzed with output signals related to the type and concentration of pathogen in the medium. The SGAW biosensors offer rapid, real-time, label-free analyses that are claimed to be cost effective and easy to use although they are not currently available commercially and some can require long incubation times of the bacterial sample on the biosensor surface [103]. At present, reports on these systems are limited, so they do not address potential issues such as diffusion and detection of pathogens in large volumes of water.

#### 5.1.5. RAPTOR Fiber Optic Biosensors

The RAPTOR fiber optic biosensor is a type of EWFOS that monitors complex formation by evanescently exciting surface-bound tracer antibody fluorophores with a diode laser [98,104]. The assay is achieved in less than 10 min in a fully automated, portable unit with few moving parts and long-lasting probes. This system has been successfully demonstrated in food safety applications [105] and for the detection of pathogens in irrigation and recreational waters [106,107]. A major limitation of the system is the need for replacement of the fiber probes and fluorescent reagents as soon as a positive result is obtained, although up to four antibody probes can be used to detect multiple pathogens [104].

#### 5.1.6. Block II Chemical Biological Mass Spectrometer

The block II chemical biological mass spectrometer (CBMS) is an integrated system that can identify chemical and biological contaminants in a single unit [108]. The US military developed the CBMS, that is similar to the RAPTOR biosensor system, primarily for the detection of threats and as such, the instrument is portable and robust. The CBMS system can only use a single detection mode at any time but the instrument has the ability to rapidly switch between detectors. This detection system has potential as a membrane integrity device but it is not currently commercially available.

#### 5.1.7. Miniaturized Portable Biosensors

This technique uses a miniaturized gold electrode biosensor to detect pathogens by immobilization of antibodies onto the bio-functionalized electrode. An electrochemical technique based on impedance spectroscopy, the test can take several hours to complete and is currently only being developed for clinical purposes [109]. At present the device has been developed to detect different strains of the avian influenza virus but it has the potential for further development to detect other similar viruses. However, the data acquisition time and the technical capacity to operate and maintain the instrumentation limits its application in the field for membrane integrity surveillance.

#### 5.1.8. Microarray Biosensors

A microarray biosensor uses an automated concentration system to detect pathogens in water using a range of detectors including fluorescence [110] and other optical techniques [111]. Systems can be configured for online integration and although offering rapid analyses, these tests generate minor quantities of waste products [112]. Moreover, the detection limits of typical bacteria are relatively high on the order to 10^5^ CFU/mL for *E. coli*, so it is unlikely that high LRVs could be achieved with these sensors.

#### 5.1.9. Surface Plasmon Resonance Biosensors

Surface plasmon resonance (SPR) biosensors offer qualitative and quantitative label-free detection and analysis of biomacromolecules including proteins and nucleic acids in real-time [113,114,115,116]. There are a wide range of configurations for various applications but the general principal of SPR biosensors is based on the change in refractive index of a metallic surface irradiated with a light source. Light is reflected at an angle defined by the type and amount of target bacteria in the solution that are bound to the metallic surface which is then measured and quantified. Although most configurations are capable of detecting large molecules, some sandwich assays and competitive inhibition assays can detect much smaller molecules including engineered nanoparticles [117,118] and the presence of gold nanoparticles can also enhance SPR signals [113]. Utilizing this technique in the field for membrane system integrity surveillance may be challenging due to environmental exposure of the sensitive instrumentation.

#### 5.1.10. Quantum Dot Based DNA Nanosensors

These ultrasensitive nanosensors use quantum dots (QDs) linked to DNA probes to capture and concentrate DNA from specific targets. The detection is based on fluorescence resonance energy transfer (FRET) to detect DNA at low concentrations which is capable of generating a distinct, highly detectable FRET signal [119]. Single QD-based sensors have also been developed to detect multiple virus strains in a single assay [120]. Although typically used for clinical tests, there is a potential for other applications such as direct integrity monitoring for pathogen transport through membrane systems. The system requires specialized personnel to operate the bench-scale systems and it is a relatively expensive technique that currently limits the potential application in membrane integrity surveillance. Similar to other engineered nanoparticles, solubility, surface chemistry and the potential for agglomeration are also potential issues for QDs.

#### 5.1.11. Laser Scanning Cytometry

Online laser scanning cytometry (LSC) can be used to detect and enumerate microspheres in feed and permeate samples [121]. The LSC technique is based on fluorescence using a fixed laser light source but on a fixed membrane sample rather than in a flowing solution such as a permeate sample. At present the technique is only applicable to micron sized particles using a bench-scale prototype instrument, although there is a strong potential to develop more sensitive, portable instruments providing the costs can also be reduced.

#### 5.1.12. Microfluidic Biochip Systems

Microfluidic systems for detecting pathogens cover a range of miniaturized devices utilizing techniques to measure DNA, protein and pathogen cells in integrated biochip devices [122]. Microfluidic biochips can utilize the detection systems of a range of techniques including RT-PCR for DNA analysis, protein/enzyme sensing and cell-based assays to identify and quantify different cellular systems. In general, these systems offer high surface to volume ratios in microchannels which increases the probability of pathogen interaction and cell capture at the sensor surfaces allowing rapid identification of small amounts of pathogens [104]. A wide range of commercial examples of these systems are available that can target different pathogens such as viruses and bacteria [122]. Rapid developments in technologies such as these could ultimately lead to developing these systems for membrane integrity monitoring [104], however, there have been no recent developments to further the prospects for this technique for membrane integrity surveillance.

### 5.2. Other Detection Systems

Developments in nanotechnologies over the past few decades have required new techniques for the detection of synthetic nanosized particles in a range of media [123,124,125]. Most viruses and many bacteria fall within the nanometer size range so techniques used to detect nanoparticles in water are readily applicable to NF and RO integrity monitoring, particularly where high sensitivity at low concentrations are available. The ability to detect single virus particles in a solution is perhaps the ultimate challenge for a membrane integrity test and there are several recent attempts to achieve this goal.

#### 5.2.1. NanoSight Particle Tracking

The NanoSight nanoparticle tracking analysis (NTA) system was first commercialized in 2004 and covers a range of biological and biochemical applications [125]. Not unlike conventional zetasizer instruments, the NTA system can measure the particle size and zeta potential of nanoparticle suspensions. In addition to these analyses, the NTA system can measure fluorescence and the concentration of particles in solution in the size range 10–2000 nm diameter [126]. Particle counting relies on the diffusion of particles and subsequent micrograph imaging and counting which is sensitive to camera settings [127]. A report of the application of NTA technologies details a wide selection of examples of the versatile system in use, including uses in virology and vaccines, nanoparticle systems and drug delivery [125]. The potential for scaling the system to a robust, portable, online instrument is not clear and this may limit its use to offline assays at present.

#### 5.2.2. Online Chemical Oxygen Demand

AquaDiagnostic Pty Ltd., Melbourne, Australia, produces an online system to measure the COD of water samples [128]. Results can be obtained in less than 5 min using a robust, portable instrument that can perform a direct measurement of absolute COD that avoids the need for calibration. The minimum detection limit is reported to be 0.2 mg/L with a working COD range up to 350 mg/L but some consumables and reagents are required. Table 3 shows results of a test performed using the COD analyzer on the first and second pass RO permeates from a water recycling plant in Victoria, Australia. The COD results clearly show the sensitivity of the technique to measure low levels of organics and the test was also able to demonstrate a significant difference between the first- and second-pass RO membranes. Although the COD of the RO feed was not measured, the RO feed had a TDS of 3160 mg/L so the 2nd pass permeate had an apparent LRV of 3.1.

The challenge for the online COD technique is to differentiate between chemical compounds and biological material that both exert a chemical oxygen demand. The data provided by the COD instrumentation is similar to that provided by the more expensive TOC instrument and has similar limitations based on the vagaries of feed concentration and the interferences and inhibitions arising from heavy metal contamination and chloride ion concentrations.

#### 5.2.3. Whispering Gallery Microlasers

Whispering gallery mode (WGM) microlasers are a type of evanescent wave sensor that detects discrete changes in the WGM resonance frequency excited in a microspherical cavity. The resonance shift is detected in response to the presence of a bound single virus particle such as the influenza virus [129]. The WGM concept is based on the observation that sound waves could travel around a concave surface first discovered in the late 1800’s in the whispering gallery of St Paul’s Cathedral [130]. The relatively recent development of WGM microresonator systems based on this acoustic phenomenon offer label-free detection of single viral pathogens but is currently only available in bench-scale systems [131,132,133]. In addition, only very small volumes can be measured so this may limit its applications to low throughput applications.

#### 5.2.4. Fluorescence Emission Excitation Spectroscopy

**Figure 4:**
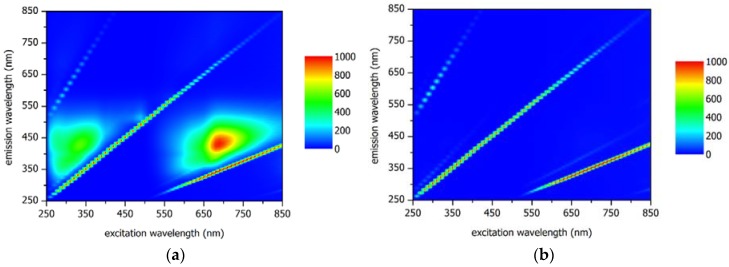
Examples of EEMs from RO: (**a**) feed and (**b**) 1st pass permeate.

Typical fluorescence emission excitation matrix (EEM) spectroscopy scans present emission fluorescence signals as a function of incremental changes in excitation wavelengths [134]. The result is a three dimensional, or contour, plot which highlights areas of high fluorescence intensity [135]. In water and wastewater samples, these areas are related to dissolved organic matter (DOM) with specific regions attributed to distinct components including proteins and organic acids. Fluorescence EEMs are widely used to monitor water quality [136,137,138,139,140], to track effluent flows [141,142], and to monitor recycled water schemes [140,143,144,145]. In most cases, qualitative data is obtained but in some cases, semi-quantitative data can be obtained and changes in organic species can be monitored [146]. Figure 4 shows examples of EEMs from the RO feed and first stage RO permeate from a water recycling plant in Victoria, Australia. The RO feed clearly shows regions of fluorescent organics (Figure 4a) that are not present in the first stage RO permeate (Figure 4b).

Some reports have claimed the use of fluorescence EEMs to characterize RO permeates from multiple pass systems [147]. In this report, qualitative and quantitative analyses of EEMs could distinguish between the different stages with a general trend towards lower organic fluorescence across the stages. Recently, a direct evaluation of the use of fluorescence EEMs for RO integrity monitoring compared the technique to conductivity testing [39]. The results outlined a procedure for analyzing the EEMs and the results suggest that the fluorescence technique is more sensitive than conductivity testing with DOM rejections of >99.9% obtained or better than 3 log_10_ removal.

Quantifying the capacity to reject DOM can be used to infer the integrity of a membrane system and will generally understate the case as low molecular weight DOM can pass through a high-pressure membrane where virus-sized particles will be rejected leading to a more conservative LRV for the system being tested. The ability to identify the log removal potential of a membrane system using EEM is suitable where DOM is the principle measure, however, where nanoparticles such as viruses are the primary concern, then this technique provides an inferred, qualitative surveillance capacity.

#### 5.2.5. Quantum Dots

Many types of QDs are reported to be highly detectable with strong fluorescence at low concentrations [148,149,150,151,152,153]. Typical QDs are formed using cadmium or zinc with the latter preferred due to its lower toxicity to humans and the environment [154]. Due to their high sensitivity and their ease of synthesis, QDs could potentially be used for challenge tests providing the size of the particles could be extended beyond the relatively small sub-10 nm range to virus-sized particles on the order of 20 nm [155].

More recently, a new class of QD has emerged based on carbon. Nano-diamonds or carbon QDs are reported to be biocompatible, highly fluorescent, easy to synthesize and can be formed in a range of sizes [156,157,158,159,160,161,162]. These materials are now finding applications in the medical field as tracers and biomarkers [163,164] and due to their apparent compatibility with biological systems, they may offer a new type of safe, highly sensitive membrane integrity challenge test. The use of nano-diamonds to detect the presence of brain tumors in humans has been reported by researchers at Sydney University [165] where targeted drug delivery methods to destroy tumor growths are being developed. Although the technique is still primarily focused on human therapies, it has potential to monitor membrane integrity as the nano-diamonds are reported to be relatively inexpensive to synthesize. However, this report was on the basis of medical applications which may be comparatively expensive for the water industry where large quantities of surrogates are required for challenge tests.

## 6. Assessing the Current and Emerging Techniques

Table 4 presents a summary of current and emerging techniques for NF/RO membrane integrity monitoring. The advantages and limitations are presented with a brief description of the test and the general application of the test to membrane systems. It is evident from this review that there are numerous techniques that are already available to be fully explored as options for online integrity monitoring and others that could be further developed. However, the limitations reported and the complexity of some of the techniques reduces the opportunities considerably. The membrane integrity techniques shaded in grey are deemed to adequately meet most or all the objectives of an ideal integrity test described earlier. Dye and TRASAR^®^ testing rely on the detection of optically active chemicals that can potentially be detected at ng/L levels, particularly the fluorescent dyes including RWT, fluorescein and TRASAR^®^. Instrumentation for the detection of these dyes is readily available and the capacity for implementation at full-scale and in real-time is high and at relatively low cost. Similarly, and perhaps closer to the ideal surrogate, fluorescent nanospheres are another promising and viable option, although the costs of synthesizing the required quantities of these particles for full-scale testing may be prohibitive. Moreover, the fate of synthetic nanomaterials in the reject water is relatively unknown.

## 7. Conclusions

The techniques identified in this review are widely varied based on their sensitivity and the capacity to perform the tests in real-time and at a reasonable cost. Of all the methods, those based on challenge testing are among those that would be more acceptable to industry based on the availability of the surrogate species and the ease at which the tests can be implemented. Fluorescent chemical compounds and tagged nanoparticles are becoming more widely accepted as alternatives to the MS2 bacteriophage as they show the required sensitivity to provide an LRV performance up to and potentially beyond 4 log_10_. A range of new and innovative techniques based on the detection of individual pathogens or the use of sensitive detection equipment also show some promise for the future development of novel integrity monitoring techniques.

## Figures and Tables

**Table 1:** Suggested criteria for ideal integrity monitoring systems or devices.

Criteria	Requirement(s)
*Technique Considerations*	
Test type	Test should be online and provide integrity performance (LRV) results in real-time. Detection in permeate from membrane elements, individual pressure vessels or membrane array of complete membrane train. System shutdown unnecessary, normal operations continue during integrity surveillance.
Sensitivity	High sensitivity at low challenge species concentration.
Selectivity	Challenge species should be representative of the smallest virus rather than chemical compounds and not be subject to changes in detection resulting from variations in environmental or chemical conditions such as NOM, salinity, pH and temperature.
Output	Test should deliver minimum LRV of 4 log_10_ sensitivity.
*Financial Considerations*	
Capital cost	In the same order or less of capital cost as existing online real-time systems such as total organic carbon instrumentation.
Installation Integration	The ability to be fully integrated into existing systems as well as new systems seamlessly (greenfield and brownfield applications).
Operation	Should require minimal training for operators.
Running costs	Should not add more than 1–1.5% of the tariff charged to consumers for the provision of treated water.

**Table 2:** Summary of reported RWT integrity tests.

Feed Concentration (mg/L)	Mode *	LRV	Reference
0.1–1	C	3.5–5.3	[54]
1–2	C	3.9	[8]
1	C	2.7–3	[9]
0.1–1	C	2–5	[57]
0.1	C	2.6	[56]
5–10	P	>4	[58]

* Mode: C = continuous, P = pulse.

**Table 3:** Example of TDS and COD results of 1st and 2nd stage RO permeates.

RO Permeate	TDS (mg/L)	COD (ppm)
1st pass	48.2	1.57
2nd pass	2.4	0.27

**Table 4:** Assessment of current and emerging integrity monitoring methods.

Monitoring Technique	Membrane Applications	Mode	Description	Scale	Advantages/Limitations	References
***Existing Techniques—Direct Monitoring***						
Vacuum Decay Testing	NF and RO membranes	Offline	Element soaked with RO permeate overnight, drained then capped, vacuum applied; decay monitored over 1 min; fail at >10 kPa/min decay	Post-manufacturing; bench- and pilot-scale	Applies only to individual elements and not to the entire system	[6,9,28,71]
Pressure Decay Testing	MF, UF, NF and RO membranes	Offline	One side of the membrane pressurized, pressure loss over time monitored	Bench- and pilot-scale; can be used for entire stage of NF and RO systems	Not practical for full-scale elements due to drainage requirement; pressurizing permeate side can cause damage to NF/RO membrane; not widely used for these systems	[28,33,71,166]
***Existing Techniques—Indirect Monitoring—Naturally Occurring Substances***						
Particle Monitoring	MF and UF membranes	Online	Particle concentration measured in feed and permeate	Pilot-scale	Not suitable for NF/RO as particle size is too large; resolution dependent on particle concentration in feed water	[33]
Turbidity Monitoring	MF and UF membranes	Online	Similar to particle monitoring, concentration measured in feed and permeate	Full- and pilot-scale	Minimum particle size is 1 µm; low resolution	[36]
TOC Monitoring	NF and RO membranes	Online	TOC concentrations measured in feed and permeate	Full-scale; can be used for entire stage of NF and RO systems	Used in several installations but equipment to detect very low levels is expensive	[18,41]
Sulphate Monitoring	NF and RO membranes	Offline	Sulphate concentrations measured in feed and permeate	Full-scale; can be used for entire stage of NF and RO systems	Expensive to monitor continuously using ICP	[18]
Conductivity Monitoring	NF and RO membranes	Online	Conductivity of feed and permeate monitored	Bench-, pilot-, and full-scale; can be used for entire stage of NF and RO systems	Low resolution; removal limited to 2 log_10_ for water reuse applications; probing more effective than online monitoring	[41]
Periodic Testing	NF and RO elements, trains	Online	Can involve multiple tests including conductivity probing and UV-254	Full-scale of NF and RO systems	Offers multiple, periodic testing; can locate defects but is complex to implement in full scale applications	[41]
***Existing Techniques—Indirect Monitoring—Challenge Tests***						
Dye Testing	NF and RO membranes	Online	Log removal of dye measured by calibrated absorbance or fluorescence at optimum wavelength	Pilot- and full-scale	Can provide up to 4 log_10_ resolution; fouling can be an issue for some dyes but not RWT	[18,33]
Spiked Integrity Monitoring	MF and UF membranes	Online	PAC particles injected in feed side and particle concentration measured in permeate	Full-scale	Applicable only for micron size particles	[59]
Pulse Integrity Test	NF and RO	Online	Measures a pulse of highly rejected species (i.e., sulphate)	Pilot scale	Can locate defects if calibrated	[60]
Microbial Surrogates (i.e., MS2, E. coli etc.)	MF, UF, NF and RO membranes	Offline	High concentrations of surrogate introduced into feed and concentration measured in permeate	Pilot- and full-scale	Seeding required since MF/UF pretreatment will remove most surrogates; can be expensive	[71]
Fluorescent Microspheres	MF and UF membranes	Offline	Microsphere concentration in feed and permeate measured by fluorescence	Pilot- and full-scale	Up to 4 log_10_ removal reported; expensive due to cost of particles	[18]
***Existing Techniques—Integrated and Multi-Parameter Monitoring Systems***						
TRASAR^®^	NF and RO membranes	Online	Fluorescent molecules injected with antiscalant; fluorescence measured in permeate using trace leak detection	Full-scale; can be used for entire stage of NF and RO systems	Up to 6 log_10_ removal reported with non-continuous spikes; up to 2 log_10_ when used with antiscalant	[18]
Small Sensor Cell with Collection Membrane	MF and UF membranes	Online	Microsieve sensor membrane placed in permeate side stream; change in TMP of sensor membrane detects breach	Bench- and pilot-scale	Can take >60 min to detect very small breach	[83]
Binary Gas Integrity Test	MF and UF membranes	Online	Diffusivity of low permeating gas detected in permeate using mass flowmeters and composition with FTIR	Bench-scale	Complex to implement in larger membrane systems; gas permeability may be an issue as would the cost of inert gases required	[84]
ZAPS LiquID Station	General water quality monitoring device; could be applicable for MF, UF, NF and RO	Online	Measures multiple optical parameters simultaneously	Full-scale	Can potentially report high LRVs for TOC and BOD; difficult to quantify system LRV as it uses tryptophan, a common amino acid in many proteins not unique to E. coli and that may limit its sensitivity	[86]
***Emerging Techniques—Pathogen Detection Systems***						
BioSentry Device	General water quality monitoring device; could be applicable for MF, UF, NF and RO	Offline	Multi-angle light scattering at 660 nm used to determine particle size, shape and internal structure	Bench-scale	Valid only for particles greater than 0.4 micron	[96]
Real-Time Polymerase Chain Reaction	Water quality monitoring specifically for viruses	Offline	Feed and permeate collected and virus detected using centrifugation, filtration and enumeration techniques	Bench-scale	Requires specialized personnel, sample preparation and long time periods for results; expensive	[97]
Evanescent Wave Fiber Optic Sensor	Detection of pathogens	Online	Laser derived evanescent wave is excited over sample and fluorescence measured using laser spectrofluorometer	Bench-scale	Long detection time (several h)	[98,99,100]
RAPTOR Fiber Optic Biosensor	Detection of pathogens	Online	Monitors complex formation by evanescently exciting surface-bound fluorophores with a diode laser	Bench-scale	Portable; results in less than 10 min	[104]
Miniaturized Portable Biosensor	Detection of pathogens	Online	Electrochemical technique (impedance spectroscopy) used to detect virus by immobilization of antibodies onto biofunctionalized gold electrode	Bench-scale	Long detection time (several h)	[109]
Microarray Biosensor Instrument	Detection of pathogens	Online	Automated concentration system uses advance array biosensor to detect pathogens in water	Bench-scale	Laboratory-scale systems common	[112]
Surface Plasmon Resonance Biosensors	Detection of pathogens	Online	Illumination of a metallic surface by visible or near-infrared radiation from a monochromatic light source via a hemispherical prism; electromagnetic waves are generated and detected	Bench-scale	Not currently available as a commercial technique for field applications	[116]
Quantum Dot Based DNA Nanosensors	Detection of pathogens	-	Ultrasensitive nanosensor based on fluorescence resonance for detecting DNA	Bench-scale	Requires specialized personnel; expensive	[119]
Laser- Scanning Cytometry	Detection of pathogens	Online	Laser-scanning cytometry used to detect microspheres in feed and permeate samples	Bench-scale	Only applicable for micron-sized particles	[121]
